# Regulation of voltage-gated sodium channel expression in cancer: hormones, growth factors and auto-regulation

**DOI:** 10.1098/rstb.2013.0105

**Published:** 2014-03-19

**Authors:** Scott P. Fraser, Iley Ozerlat-Gunduz, William J. Brackenbury, Elizabeth M. Fitzgerald, Thomas M. Campbell, R. Charles Coombes, Mustafa B. A. Djamgoz

**Affiliations:** 1 Neuroscience Solutions to Cancer Research Group, Department of Life Sciences, Imperial College London, South Kensington Campus, London SW7 2AZ, UK; 2 Department of Biology, University of York, Heslington, York YO10 5DD, UK; 3 Faculty of Life Sciences, University of Manchester, Manchester M13 9PT, UK; 4 Department of Cancer and Surgery, Faculty of Medicine, Imperial College London, Hammersmith Campus, Du Cane Road, London W12 0NN, UK

**Keywords:** voltage-gated sodium channel, metastasis, activity-dependent regulation, growth factor, hormone

## Abstract

Although ion channels are increasingly being discovered in cancer cells *in vitro* and *in vivo*, and shown to contribute to different aspects and stages of the cancer process, much less is known about the mechanisms controlling their expression. Here, we focus on voltage-gated Na^+^ channels (VGSCs) which are upregulated in many types of carcinomas where their activity potentiates cell behaviours integral to the metastatic cascade. Regulation of VGSCs occurs at a hierarchy of levels from transcription to post-translation. Importantly, mainstream cancer mechanisms, especially hormones and growth factors, play a significant role in the regulation. On the whole, in major hormone-sensitive cancers, such as breast and prostate cancer, there is a negative association between genomic steroid hormone sensitivity and functional VGSC expression. Activity-dependent regulation by positive feedback has been demonstrated in strongly metastatic cells whereby the VGSC is self-sustaining, with its activity promoting further functional channel expression. Such auto-regulation is unlike normal cells in which activity-dependent regulation occurs mostly via negative feedback. Throughout, we highlight the possible clinical implications of functional VGSC expression and regulation in cancer.

## 1. Introduction

It is now well established that de novo expression of voltage-gated ion channels (VGICs) occurs in cancers *in vitro* and *in vivo* and plays a significant role in disease initiation and progression [1–6]. In particular, voltage-gated Na^+^ channels (VGSCs) are functionally expressed in many types of carcinomas (cancers of epithelial origin), including those of breast, cervix, colon, lung (small-cell, non-small-cell and mesothelioma), skin, ovary and prostate, where they promote disease progression, potentially leading to metastasis [7]. On the other hand, voltage-gated K^+^ channels (VGPCs) commonly control cellular proliferation in which VGSC activity plays no role [8,9]. In addition, VGPCs may be downregulated as cancer becomes more aggressive [2,8]. Such dichotomy in VGSC–VGPC expression in cancer is consistent with the notion that primary and secondary tumours are associated with expression of different genes and can even be controlled independently [10].

**Figure 1.**
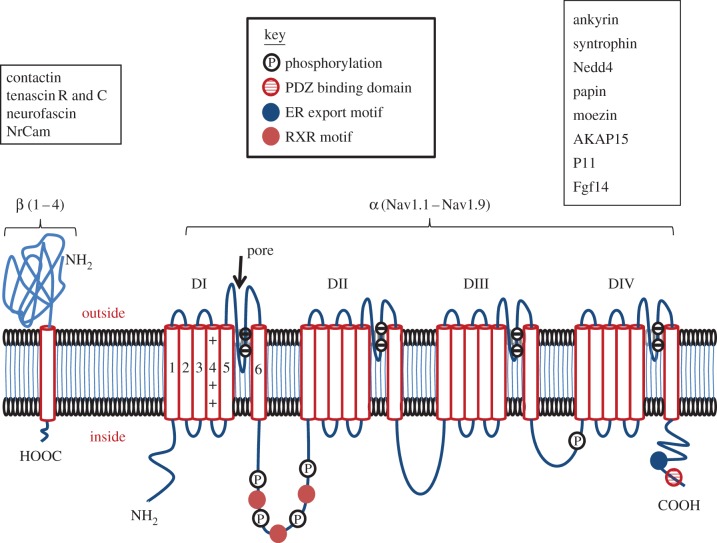
Schematic diagram of the structure and membrane topology of the voltage-gated sodium channel showing the main regulatory sites. Given α-subunits have four domains (DI–DIV) each composed of six transmembrane segments. Within the latter, segment four contains positively charged amino acids and this is the main voltage-sensitive region; the loop between transmembrane segments 5 and 6 is negatively charged and forms the pore region. Many types of modulatory sites exist for both α- and β-subunits as indicated by the key. The boxes adjacent to α- and β-subunits list the proteins known to interact with each subunit, respectively. PDZ, post-synaptic density protein (PSD95) and *Drosophila* disc large tumour suppressor (Dlg1) and zonula occludens-1 protein (zo-1); ER, endoplasmic reticulum; RXR, a motif which mediates retention of proteins in the ER. (Online version in colour.)

Expression and/or activity of VGICs can be regulated from transcription to post-translation. These include specific mechanisms of post-transcription/pre-translation, e.g. miRNAs [11] and post-translation, e.g. intracellular trafficking [1,2,6]. The primary regulators include hormones (mainly steroids and peptides) and growth factors which, in fact, are all intimately associated with various aspects and stages of the cancer process. In addition, some drugs used for cancer treatment may affect VGICs [12,13]. Here, we focus on the regulation of VGSCs which comprise a multi-gene family of at least nine different functional members (Nav1.1–1.9) coding for the pore-forming α-subunits (figure 1) [14]. There are also four auxiliary β-subunits of which one or two at a time can associate with an α-subunit and modulate channel expression and activity in plasma membrane [14].

Several individual Nav isoforms are known to be functionally expressed in different human cancers. These include Nav1.5 in breast and colon cancers [8,15,16]; Nav1.6 in cervical cancer [17,18]; and Nav1.7 in breast, prostate and non-small cell lung cancers [8,19,20]. In addition, where studied (especially for Nav1.5 and Nav1.7), alternative splice variants of the isoforms have been found with sequence differences most notably in the extracellular loop between segments 3 and 4 of domain I [8,19,21]. At present, the rationale for such a phenomenon to occur in cancer is unclear, but consistent with channel expression being both (i) epigenetic [22] and (ii) oncofetal [23]. The mechanisms controlling alternative splicing also are not well characterized but could involve cAMP [24] and activity-dependence [25].

In the case of breast cancer (BCa), the oncofetal/neonatal isoform of Nav1.5 (nNav1.5) would allow greater Na^+^ entry into the cell, compared with the adult splice variant [26]. In turn, this could have implications for Na^+^–H^+^ exchange and intra/extracellular pH regulation [27]; enzyme activity, e.g. Na^+^/K^+^-ATPase and protein kinase A [27,28]; and Ca^2+^ homeostasis, e.g. Na^+^–Ca^2+^ exchange [29]. In addition, the charge-reversing aspartate (negative) to lysine (positive) amino acid change that occurs at position 211 could affect (i) response to extracellular (intrinsic or extrinsic) chemical factors, e.g. pH [30] and (ii) protein–protein interactions [31].

VGSC activity has been shown to contribute to many cell behaviours that may be important for metastasis. These include migration [32–34]; invasion [8,15,16,18,20,34–38]; colony formation in three-dimensional Matrigel [39]; process extension [40]; galvanotaxis; [8,41]; adhesion [42]; gene expression [43]; endocytic membrane activity [44–46]; vesicular patterning [47]; nitric oxide production [48]; and invadopodia formation [49]. Although the mechanisms underlying the involvement of VGSCs in such cell behaviours are not well characterized, several suggestions have been made. These include (i) interaction with cytoskeletal elements (and/or β-subunits) and (ii) modulation of ion fluxes/exchangers, gene expression and enzyme activity [8,18,32,39,49,50]. However, only a few specific mechanisms have been shown directly to be involved in the proposed role of VGSCs in metastasis-associated cellular behaviours. These mechanisms include PKA [51] and Na^+^/H^+^ exchanger [39,49]. Importantly, also, *SCN5A* (the gene that encodes Nav1.5) was deduced to be upstream of a network of genes/signalling cascades controlling human colon cancer invasiveness [16]. Thus, VGSCs would appear to play a significant role in cancer progression and are a potential novel therapeutic target against metastasis, the main cause of death in cancer patients [5,7,8,52].

Our overall approach here is to review, wherever possible, the available evidence for regulation of VGSC expression/activity in cancer cells. However, for completeness, we also cover some essential non-cancer examples.

## 2. Hormones

**Figure 2.**
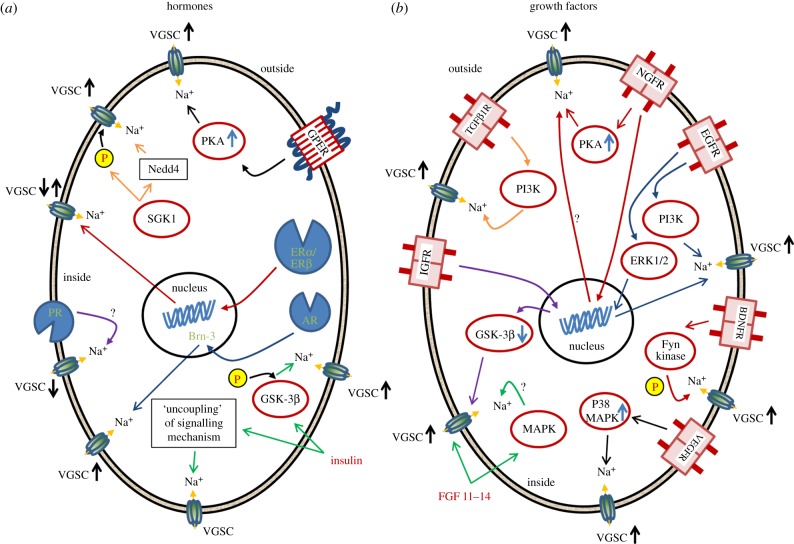
Summary diagrams showing possible regulatory pathways controlling voltage-gated sodium channel (VGSC) expression/activity in cells by (*a*) hormones and (*b*) growth factors. Some pathways mediate transcriptional activity; some actions are through the respective receptors, whereas some actions are directly on the channel. Some of the regulatory pathways have only been described in normal cells. Abbreviations are defined in text. VGSC↓ and VGSC↑ denote decreased and increased VGSC expression/activity, respectively. (Online version in colour.)

The development and/or progression of many cancers are well known to be hormone-dependent; hormone independence may develop during treatment, whereupon the cancer may become more aggressive [53]. Some cancers show particularly strong hormone sensitivity owing to the inherent nature of the native tissue, as for BCa and prostate cancer (PCa) for which oestrogen and androgen are key steroid hormones, respectively. Consequently, such cancers are commonly treated with hormone-based medication. A wide range of signalling mechanisms and cascades are associated with hormone action upon VGSCs, as illustrated in figure 2*a*.

### (a) Oestrogen

The effects of β-oestradiol (E2), the biologically active form of oestrogen, are classically mediated by two types of oestrogen receptor (ER): ERα and ERβ, which belong to the superfamily of ligand-activated transcription factors (‘nuclear receptors’). Like other members of this group, ERs function as dimers, which when bound to their ligand, undergo a translocation from the cytoplasm to the nucleus where they recognize specific sequences of DNA-response elements in the promoter regions of the target genes, ultimately resulting in their up- or downregulation [54]. However, non-transcription modes of ER action also occur. Thus, ERα can be localized at the plasma membrane in multi-protein complexes and mediate fast, non-genomic effects; several possible variants of such receptors exist including ER46, ERα-36. In addition, a novel transmembrane G-protein-coupled ER (GPER), previously called ‘GPR30’ also mediates fast, non-genomic effects [55].

**Figure 3.**
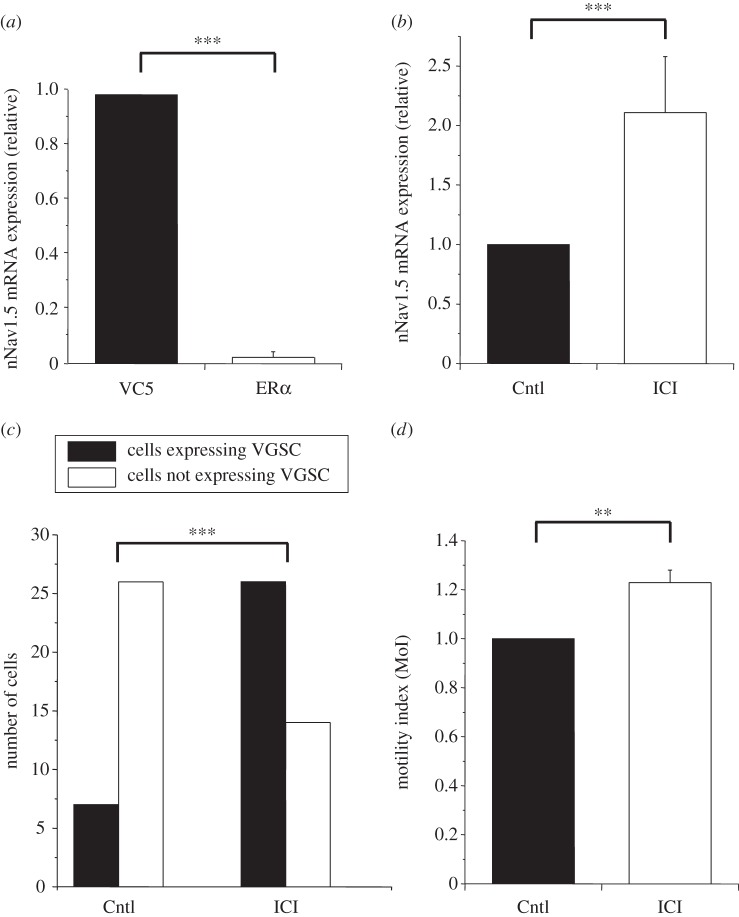
Effects of oestrogen receptor α (ERα) on voltage-gated sodium channel (VGSC) expression and activity in MDA-MB-231 cells transfected with ERα (MDA-MB-231-ERα cells). (*a*) Basal levels of nNav1.5 mRNA were significantly lower in MDA-MB-231-ERα cells (ERα) compared with control cells expressing only the plasmid vector (VC5; *p* < 0.001; *n* = 4). (*b*) Treatment of MDA-MB-231-ERα cells with the ER antagonist ICI-182,780 (ICI; 1 µM) for more than 72 h significantly increased the nNav1.5 mRNA level, compared with non-treated cells (Cntl; *p* < 0.001; *n* = 4). (*c*) Similar treatment with ICI-182 780 for more than 48 h significantly increased the number of cells with VGSC activity, in comparison with those grown in normal medium (5% FBS–Cntl) as determined by patch-clamp recording (Fisher's exact test: *p* < 0.001; *n* = 73 cells). (*d*) Treatment of MDA-MB-231-ERα cells with ICI-182,780 for more than 72 h significantly increased the lateral motility of the cells (*p* < 0.01; *n* = 4). Further information is given in the supplementary Methods section.

A basal ‘negative’ association between expression of ERα and VGSC is apparent in human BCa cells. Thus, the strongly metastatic MDA-MB-231 cells are devoid of classic ERα, but express a functional VGSC, in particular nNav1.5 [8]. Conversely, the weakly/non-metastatic MCF-7 cells are ERα-positive and do not express any functional VGSC [8]. Additional data were obtained from MDA-MB-231 cells stably transfected with functional ERα (MDA-MB-231-ERα cells) [56]. In these cells, compared with controls expressing only the plasmid vector (VC5), there was a significant decrease in nNav1.5 mRNA levels by 96 ± 2% (figure 3*a*). Consistent with this, the proportion of cells expressing functional VGSCs (nNav1.5) was reduced from 71 to 43% (*p* < 0.05; *n* = 10–15 cells) [57]. Furthermore, treatment of the MDA-MB-231-ERα cells with the ER antagonist ICI-182 780 for more than 48 h produced the following significant effects: (i) the nNav1.5 mRNA level was increased by 211 ± 49% (figure 3*b*); (ii) the proportion of cells expressing functional VGSCs was increased by more than three-fold (figure 3*c*); and (iii) the lateral motility of the cells was increased by 23 ± 5% (figure 3*d*).

Taken together, these results suggest that steady-state VGSC expression is upregulated transcriptionally in the absence of ERα expression/activity, in general agreement with ERα-negative (VGSC-expressing) cases of BCa being more aggressive [58]. Comparable data were obtained from ERα-knockout mice where mRNA expression of most Nav isoforms studied was upregulated [59].

However, MDA-MB-231 cells do express a ‘cell-surface’ ER. Thus, acute (10 s), extracellular application of E2 (10 nM) increased nNav1.5 current, via GPER coupled to PKA activation, leading to a reduction in cellular adhesiveness [51]. Acute application of E2 (10 nM) also increased VGSC currents in rat hypothalamus by a non-genomic mechanism [60]. By contrast, E2 inhibited VGSC currents in cultured N1E-115 mouse neuroblastoma cells [61]. Similarly, mouse dorsal root ganglion (DRG) neuronal VGSCs were inhibited by acute application of E2 and this occurred via a cell-surface ER-activated protein kinase C (PKC)–PKA pathway [62]. Thus, the quick effects of E2 on VGSCs may involve different intracellular signalling cascades, dependent on cell type.

The functional association of ERα/E2 with VGSCs is of clinical significance, because a significant body of evidence suggests that clinically prescribed ‘selective oestrogen receptor modulators’ (SERMs) can also affect VGSCs. Thus, raloxifene (‘Evista’) inhibited the VGSC current in guinea pig ventricular myocytes [12]. Tamoxifen also inhibited VGSC activity in the SHG-44 glioma cell line [13] and in rat cortical neurons [63]. Similar effects of SERMs were seen on rodent hypothalamic neurons [64] and ventricular myocytes [65].

In conclusion, (i) E2 has both non-genomic and genomic effects upon VGSC expression/activity; and (ii) transcriptionally, E2 (via ERα) downregulates functional VGSC (nNav1.5) expression in BCa cells. Some of these effects may manifest clinically in hormone-based treatment of patients and may impact upon the treatment itself. For example, drug-induced blockage of VGSCs may impair nerve and/or muscle activity. More work is needed (i) to generalize these notions; (ii) to improve our understanding of the role of ERβ; (iii) to elucidate whether the effects differ between cancer and corresponding normal cells; and (iv) to integrate the fast non-genomic effects with long-term genomic regulation [66].

### (b) Androgen

Two isoforms of androgen receptor (AR) have been identified: AR-A, a 87-kDa protein with 187 amino acids cleaved from the amino-terminal domain and AR-B (110 kDa) which is considered the full-length receptor [54]. Little is known regarding the effects of androgen on VGSCs in cancer cells. As in the case of BCa cell lines, however, there is a negative association between basal expression of AR and VGSC in PCa cell lines. Thus, VGSC (Nav1.7) activity occurs in strongly metastatic, AR-devoid PC-3/M cells while AR-expressing, weakly/non-metastatic LNCaP cells do not possess functional VGSCs [19]. Application of (5α,17β)-17-hydroxy-androstan-3-one dihydrotestosterone (DHT) to androgen-deprived LNCaP cells decreased VGSC β1 mRNA expression over 24–48 h [67]. Surprisingly, DHT treatment also significantly (but transiently) increased both β1 and Nav1.7 mRNAs in PC-3 cells, presumably through a non-AR-dependent mechanism [67]. Such an effect could be mediated via a ‘cross-activated’ growth factor receptor signalling cascade [68]. In neuroblastoma ND7 cells, a nuclear interaction between the developmentally regulated transcription factor Brn-3a and AR resulted in a complex which bound to multiple elements within the promoter region of *SCN9A* (Nav1.7) and upregulated channel expression [69]. In differentiating mouse muscle C2 cells, VGSC currents were reduced by androgen treatment and abolished when AR was overexpressed; there was no change in VGSC mRNA level, suggesting that the inhibition was post-transcriptional [70].

In conclusion, from the limited available evidence, the effects of androgens on VGSC expression/activity appear complex. On the whole, however, the steady-state association is negative in PCa (as in BCa), again in line with hormone-insensitive (VGSC-expressing) cases of PCa being relatively more aggressive.

### (c) Other hormones

Several other cancer-associated hormones also affect VGSC expression/activity. Insulin is a peptide hormone and its receptor (InsR), a tyrosine kinase, can occur in two alternatively spliced isoforms: InsR-A and InsR-B; the former is the predominant isoform in fetal life and is also the main subtype expressed in cancer as an ‘oncofetal’ phenomenon [71]. In InsR-expressing human BCa MDA-MB-231 cells, addition of insulin (in serum-free medium) increased cellular migration [72]. Interestingly, blocking VGSC activity by applying tetrodotoxin (TTX) in the presence of insulin increased, rather than decreased, migration implying that insulin additionally controlled the functional ‘coupling’ between VGSC activity and the cells’ motility [72]. Insulin has been shown to affect VGSC expression/activity also in non-cancer cells. In cultured bovine adrenal chromaffin cells, insulin increased cell-surface expression of VGSC (Nav1.7) and β1 via PI3K; the transcriptional pathway involved phosphorylation of glycogen synthase kinase-3β (GSK-3β), and downregulation of InsR substrates 1 and 2 and Akt [73]. In cardiomyocytes, the transcription factor ‘NForkhead box O 1’, which shares conserved DNA sequences with the insulin responsive element, regulated Nav1.5 expression by directly binding to the *SCN5A* promoter and inhibiting transcriptional activity [74]. Ion channels may also be modulated by glucocorticoids [75]. As regards VGSCs, serum- and glucocorticoid-inducible kinase 1 (SGK1) upregulated Nav1.5 expressed in frog oocytes [76]. The effect could involve channel phosphorylation and ubiquitin ligase Nedd4 [76,77]. In addition, although progesterone was shown to inhibit VGSC currents in cultured N1E-115 mouse neuroblastoma cells, the high IC_50_ of this effect (17 µM) would make the result rather uncertain [61]. The VGSC current in rat striatal neurons was also inhibited by microM progesterone, and this was thought to occur via a plasma membrane receptor [78]. It would be worthwhile repeating and extending these experiments, because progesterone is known to be involved in many cancers [79].

## 3. Growth factors

Growth factors (GFs) are well known to be involved in cancer initiation and progression [80]. Consequently, GF receptors and their associated signalling mechanisms are major targets for cancer therapy [81]. In general, GFs signal via their respective receptor tyrosine kinases (RTKs) which, in humans, comprise some 20 subfamilies [80]. Binding of GF activates an RTK by inducing receptor dimerization, but some may form oligomers in the absence of the activating ligand [80]. Dimerization results in the activation of the intracellular tyrosine kinase domains, in turn, triggering signalling pathways that can include JAK/STAT, MAP kinase and PI3 kinase [80]. The signalling mechanisms and cascades specifically associated with GF effects upon VGSCs are summarized in figure 2*b*.

### (a) Epidermal growth factor

**Figure 4.**
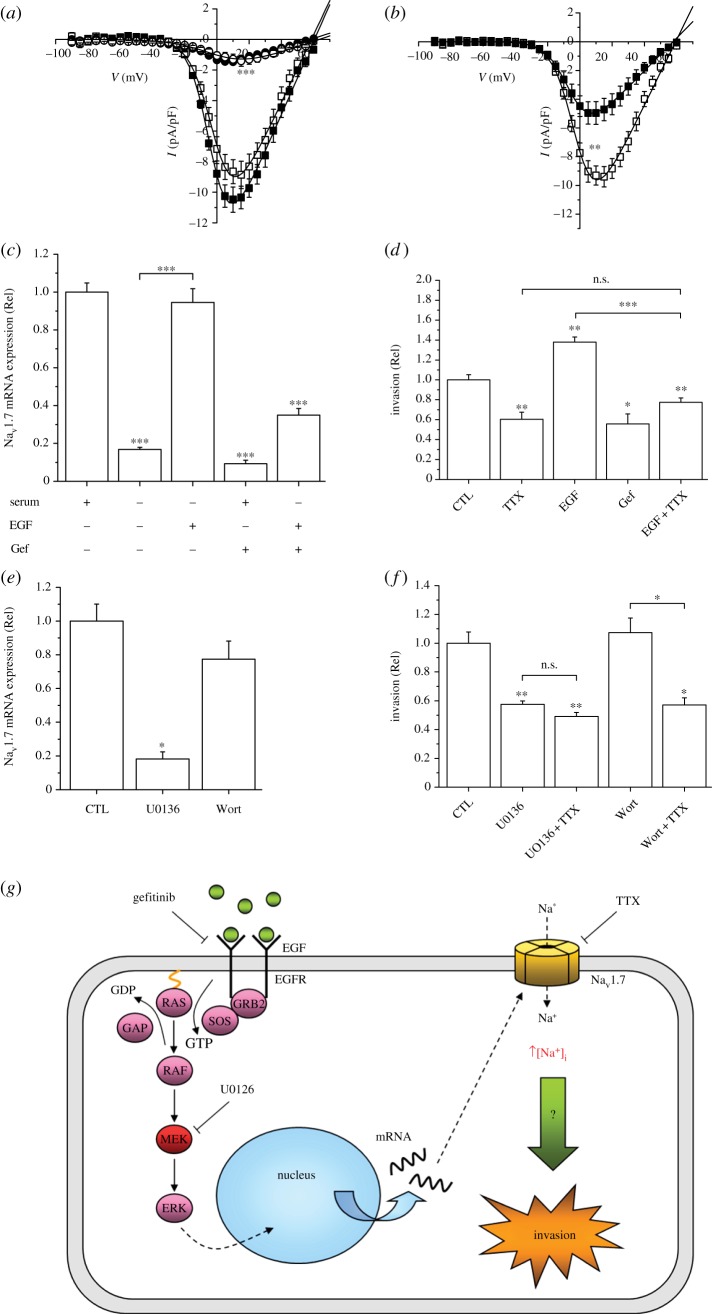
Upregulation of functional expression of Nav1.7 in human non-small-cell lung cancer H460 cells and consequent increase in invasiveness via ERK1/2 signalling. (*a*) Current–voltage (I–V) plots for control/untreated cells (open squares) and cells treated in the presence of serum for 24 h with 100 ng ml^−1^ EGF (closed squares), 1 μM gefitinib/Gef (open circles) or 10 μg ml^−1^ EGF receptor blocking antibody (filled circles). Currents were evoked using 30 ms depolarizing steps in 5 mV intervals (−90 to +70 mV) from a holding potential of −90 mV. (*b*) I–V plots for control/untreated cells (open squares) and cells treated with 10 μM U0126 (closed squares). (*c*) Relative Nav1.7 mRNA expression showing effect of serum starvation for 48 h and treatment for 24 h with EGF (100 ng ml^−1^), Gef (1 μM) and co-application of EGF + Gef. (*d*) Matrigel invasion measured after 48 h in control medium (CTL), 0.5 μM TTX, 100 ng ml^−1^ EGF, 1 μM Gef and EGF + TTX. (*e*) Effects of treatment with 10 μM U0126 or 100 nM wortmannin (WORT) for 24 h on relative Nav1.7 mRNA expression, compared with control/untreated (CTL) cells. (*f*) Matrigel invasion measured over 48 h in control/untreated cells (CTL), and following treatment with 10 μM U0126, 10 μM U0126/1 μM TTX, 100 nM WORT, and WORT + TTX. (*g*) Proposed model for EGF-mediated upregulation of Nav1.7 and consequent invasiveness of H460 cells. Stimulation of EGFR with EGF results in increased functional expression of Nav1.7 via ERK1/2. Following transcription and translation, the mature Nav1.7 protein is trafficked to the cell surface where it becomes functional. At the resting membrane potential, VGSCs are partially activated but not fully inactivated, resulting in a basal influx of Na^+^. This increase in [Na^+^]_i_ then drives cell invasion through an, as yet, unknown mechanism. All data are presented as means ± s.e. (*n* = 6–13). Statistical analyses were with Student's *t*-test or one-way ANOVA and Student–Newman–Keuls correction, as appropriate; significance: *p < 0.05, **p < 0.01, ***p < 0.001. Adapted from [89]. (Online version in colour.)

Epidermal growth factor (EGF) commonly regulates ion channel, including VGSC, expression in neurons and muscles [82–85]. In rat and human PCa cells, EGF upregulated functional VGSC (Nav1.7) expression which, in turn, promoted motility, endocytic membrane activity and invasion [86,87]. Similarly, in human BCa cells, EGF upregulated functional Nav1.5 expression through a PI3K-dependent signalling cascade [88]. In an extensive study on the human non-small-cell lung carcinoma (NSCLC) cell line H460, EGF upregulated functional Nav1.7 expression transcriptionally via ERK1/2 and increased Matrigel invasiveness (figure 4) [89]. Importantly, the EGF-induced increase in the invasiveness was blocked completely by silencing Nav1.7 expression, i.e. the effect of EGF on invasion was mediated solely via the channel upregulation [89].

In conclusion, the effects of EGF on carcinoma cell lines studied to date are consistent with the following basic scheme: EGF → VGSC upregulation (transcriptional and functional) → increased metastatic cell behaviours.

### (b) Insulin-like growth factor

Incubation of MDA-MB-231 cells for 24 h with AG1024, an inhibitor of the insulin-like growth factor-1 receptor (IGF-1R) had no effect on the VGSC (nNav1.5) peak current density [90]. In addition, in cultured rat hippocampal neurons, IGF-1 had no effect on the amplitude of the VGSC currents [91]. However, in cultured bovine adrenal chromaffin cells, chronic application of IGF-1 upregulated plasma membrane expression of Nav1.7; this was a transcriptional effect involving the PI3K-Akt pathway and GSK-3β inhibition [92]. Taken together, these findings suggested a positive feedback loop between Nav1.7 expression and GSK-3β inhibition. Further work is required (i) to determine the possible role of IGF-1R signalling in VGSC expression/activity in cancer cells, and (ii) to elucidate the possible overlap between the IGF-1R and InsR signalling in this.

### (c) Nerve growth factor

Long-term (more than 24 h) treatment with nerve growth factor (NGF) upregulated Nav1.7 functional expression in the strongly metastatic MAT-LyLu rat PCa cell line; acute application had no effect [93]. The action of NGF was suppressed by both the pan-tropomyosin-related receptor tyrosine kinases (Trk) antagonist K252a, and the PKA inhibitor KT5720, suggesting (i) that it was receptor mediated, and (ii) that PKA was a signalling intermediate [93]. Indeed, the *SCN9A* promoter in human PCa PC-3 cells was shown to be activated by NGF [94]. While it appears that NGF can affect both VGSC expression and cancer cell motility, further work is required to clarify the connection between the two effects and the nature of the associated receptor and downstream signalling mechanisms. The *in vivo* relevance of these findings also remains to be determined. Interestingly, it has recently been reported that progression of PCa in mice is accelerated by activity in the impinging autonomic nerves [95]. An intriguing question is whether such activity would release NGF from the nerves and thus impact upon the cancer VGSCs [96].

### (d) Vascular endothelial growth factor

Vascular endothelial growth factor (VEGF) plays a key role in angiogenesis [97] which also involves invasive cell behaviour [98]. Not surprisingly, therefore, VEGF has been shown to affect VGSC expression/activity in several cell types. Thus, VEGF increased the invasiveness of the cervical cancer cell line, ME180, by upregulating Nav1.6 expression via p38 MAPK signalling [99]. In human umbilical vein endothelial cells (HUVECs), VGSC (Nav1.5 and Nav1.7) activity promoted several kinds of angiogenic cell behaviour, including chemotaxis and tubular differentiation; Nav1.5 potentiated VEGF-induced ERK1/2 activation through the PKCα-B-Raf signalling axis, possibly through membrane depolarization, influx of Na^+^, reverse-mode Na^+^–Ca^2+^ exchange and rise in intracellular Ca^2+^ [100,101]. In cultured rat hippocampal neurons, VEGF decreased VGSC availability [102], whereas in bladder DRG neurons, VEGF upregulated VGSC expression leading to increased excitability [103]. Thus, VEGF can have mixed effects on VGSC expression/activity in various cell types, and it would be worthwhile to perform further studies on cancer cells and cancer-associated endothelia.

### (e) Other growth factors

Other GFs are also associated with cancer development and/or VGSC expression/activity, and two are worthy of highlighting. Fibroblast growth factors (FGFs) can be classified as secretory (FGF1–10 and FGF15–23) or intracellular/non-secretory (FGF11–14) [104]. At present, there are no published data on the possible effect of FGF(s) on VGSC(s) expressed in cancer cells. However, work on other cell types suggests that intracellular FGFs co-localize and interact directly with VGSCαs to produce a range of modulatory effects [105,106]. Transforming growth factor-β1 (TGF-β1) is a member of a superfamily of proteins that includes bone morphogenetic proteins, activins and inhibins. Activin A increased VGSC currents in the neuroblastoma Neuro 2a cell line [107]. In adult cardiomyocytes, TGF-β1 increased VGSC (Nav1.5) activity and, concomitantly, reduced the outward current [108]. This observation is reminiscent of the ‘cellular excitability’ hypothesis of cancer progression, i.e. concurrent upregulation of VGSC and downregulation of VGPC activities [2]. However, nearly opposite effects were seen on neonatal cardiomyocytes [109]. Such contrasting effects may be caused, at least partially, by changes in gene expression during development and may relate to the oncofetal nature of VGSC expression in cancer. It would be interesting to test the effects of TGF-β1 on expression/activity of VGSCs and VGPCs in cancer cells.

**Figure 5.**
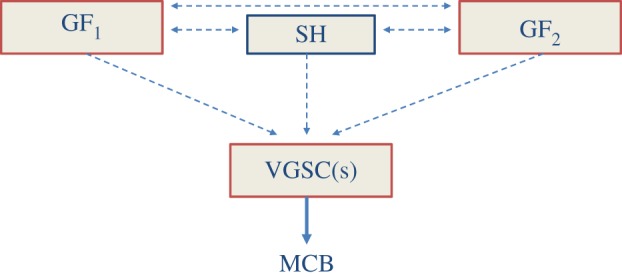
A ‘conceptual’ scheme showing how growth factors (GF_1_, GF_2_, etc.) and steroid hormone (SH) signalling systems can feed through and compensate for each other in regulating expression/activity of the VGSC(s). In turn, VGSC activity enhances metastatic cell behaviour (MCB). Dotted, horizontal lines denote the interactive pathways, involving mostly the intracellular signalling cascades. (Online version in colour.)

In overall conclusion, several GFs can affect VGSC expression and activity in a variety of cell types, including cancer and cancer-associated cells. Importantly, the multiplicity of GF regulation of VGSCs raises the possibility that it may be clinically more advantageous to block the VGSC (the ‘hub’) rather than the individual GF pathways because the latter can cross-talk (even with hormonal pathways) and compensate for each other (figure 5) [110,111].

## 4. Auto-regulation

Auto-regulation of VGSCs may occur in either of two ways: dependent upon channel activity and association with β1 subunits.

### (a) Activity-dependent regulation

Activity-dependent regulation of ion channel function is well known to occur in the central nervous system and is critical for correct neuronal development, wiring and plasticity [112]. In particular, expression of neuronal VGSCα is often regulated by negative feedback, such that patterned activity or chronic treatment with VGSC openers leads to reduction of mRNA/protein expression and change in the balance of intracellular trafficking in favour of channel internalization [113–115]. Conversely, treatment with VGSC blockers increases functional protein expression at the plasma membrane [116]. Thus, steady-state VGSC expression is normally tightly regulated in order to optimize activity and avoid hyper-excitability.

**Figure 6.**
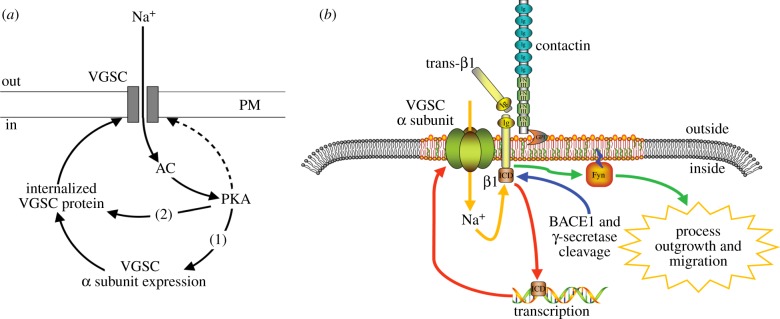
Activity-dependent regulation of VGSC expression and VGSC-dependent migration. (*a*) In Mat-LyLu [28] and MDA-MB-231 [34] cells, *I*_Na_ activates adenylate cyclase (AC) and protein kinase A (PKA), which in turn (i) potentiates α-subunit mRNA expression and (ii) increases channel expression at the plasma membrane, without affecting total cellular α-subunit protein level. PKA also directly phosphorylates surface-expressed VGSCs, although this may be independent of PKA (dashed line). Adapted from [28]. (*b*) Interplay between α and β1 subunits in transcription and process outgrowth, modelled from cerebellar granule neurons. *Trans* adhesion between β1 on an adjacent cell and a VGSC signalling complex (comprising α, β1 subunits and contactin), initiates a signalling cascade via FYN kinase that enhances process outgrowth and migration. Proteolytic processing of β1 by BACE1 and γ-secretase is proposed to release the soluble intracellular domain of β1, which may in turn enhance transcription of α subunit genes. Na_v_1.6 activity is required for β1-mediated process outgrowth, and in turn, β1 is required for normal localization of α-subunits. Thus, *I*_Na_ may fine-tune the dual processes of gene expression and migration. Adapted from [117]. (Online version in colour.)

Notably, however, a different situation appears to exist in cancer cells. In strongly metastatic rat PCa MAT-LyLu cells, expression of Nav1.7 was found to be maintained by *positive* feedback [28]. Thus, chronic pre-treatment of MAT-LyLu cells with TTX inhibited the VGSC current (*I*_Na_) which persisted after drug washout; this suppression could be reversed with the Na^+^ ionophore monensin, implying that a rise in intracellular Na^+^ was involved. In addition, TTX reduced PKA phosphorylation, and the PKA inhibitor KT5720 also reduced *I*_Na_, whereas the adenylate cyclase (AC) activator forskolin increased *I*_Na_, suggesting that the positive feedback mechanism is dependent on AC/PKA (figure 6*a*) [28,118,119]. A similar mechanism of auto-regulation was found for nNav1.5 expression in human metastatic BCa MDA-MB-231 cells [34]. Importantly, pre-treatment with TTX eliminated the VGSC-dependent migration in MAT-LyLu cells [28], and suppressed both basal and PKA-induced migration and invasion in MDA-MB-231 cells [34]. These results suggested that inhibition of *I*_Na_ would collapse the positive feedback loop, which would, in turn, reduce the cells’ migratory and invasive capacity. Indeed, it is well established that it is the influx of Na^+^ through VGSC that is essential for invasion [8,20,39]. In both BCa and NSCLC cell lines, the partial opening of VGSC at the resting membrane potential, *V*_m_, allows tonic Na^+^ influx, which in turn keeps *V*_m_ sufficiently depolarized to maintain a small but continuous influx of Na^+^ [20,39,89]. Thus, under the ensuing pathophysiological conditions, Na^+^ influx is sufficient to maintain the positive feedback and the invasive capability of cancer cells.

These data therefore suggest that in metastatic carcinoma cells, VGSCα expression is at minimum self-sustaining, and thus may potentiate metastasis in persistent fashion. The results also raise the possibility that chronic blockage of VGSCs would suppress both channel activity and expression and, consequently, provide a dual advantage in any clinical anti-metastatic treatment.

### (b) VGSC β1 subunit

Downstream functions of the VGSC β1 subunit, which is both a channel modulator and a cell adhesion molecule, may also be regulated in part by *I*_Na_ and this interaction may be reciprocal [120]. In BCa MCF-7 cells, β1 downregulation with siRNA reduced cellular adhesion to substrate and increased VGSC-dependent migration [121]. However, TTX had no effect on basal β1 subunit mRNA levels, consistent with lack of functional VGSC activity in these weakly/non-metastatic cells [121]. By contrast, the reverse could occur. Thus, downregulating *SCN1B* with siRNA upregulated nNav1.5 mRNA and protein expression [121]. It appeared that putting the cells into a higher metastatic mode by reducing their substrate adhesion automatically upregulated the VGSC expression. Such reciprocal expression of endogenous β1 and Nav1.7 has also been seen in NSCLC cells [89]. Moreover, downregulating β1 alone by siRNA was sufficient to cause a modest (approx. 30%) enhancement of A549 cell invasion, whereas overexpression of exogenous β1 reduced H460 cell invasion to a similar extent [89]. Thus, the VGSC β1 subunit has a multi-functional role in cancer and appears to be involved in a complex feedback loop that regulates channel expression/function [117]. In turn, this would modulate adhesion-mediated functions of the β1 subunit, including cellular process extension and invasiveness (figure 6*b*).

## 5. Conclusion and future perspectives

The idea that VGSCs are expressed during cancer progression and that VGSC activity enhances cell behaviours linked to metastasis, such as motility, invasion and adhesion, is now well-established. There is increasing evidence, as highlighted here, that such VGSC expression is under the control of ‘mainstream’ cancer mechanisms, principally hormones and growth factors, thus placing VGSCs as key regulators in cancer progression. However, other messenger molecules, including immune modulators, can also affect channel expression/activity [122]. Although regulation of VGSCs in cancer occurs clearly at a hierarchy of levels from transcription to post-translation, much work remains to determine the precise mechanisms involved. Regarding the former, developmentally regulated transcription factors, such as REST (‘neuron-specific silencing factor’), are also known to affect both the cancer process and channel expression [84].

It may be possible in the future, therefore, to combine conventional (e.g. hormone-based) therapies with clinically viable VGSC blockers. This may even help alleviate some of the problems associated with hormone-based therapies such as the insensitivity that frequently ensues in such treatments. Importantly, there are some indications that VGSC regulation may differ between ‘cancer’ and ‘normal’ cells. If so, understanding the signalling pathways that regulate VGSC expression/activity in cancer may provide additional avenues for preventing or suppressing metastatic disease. Furthermore, a newly discovered ‘non-canonical’ role of intracellular VGSCs in cell behaviour warrants investigation in the context of cancer [123]. In fact, a systematic analysis of the whole interactive network of ion channels and transporters is required ultimately in order to gain an overall and precise understanding of the role of ionic activity in cancer and to exploit this knowledge clinically.

## Funding statement

We acknowledge continued support from the Pro Cancer Research Fund (PCRF)—rolling grant (M.B.A.D., S.P.F.), and the Medical Research Council—Fellowship no. G1000508(95657) to W.J.B.
